# Increased Degradation Rates in the Components of the Mitochondrial Oxidative Phosphorylation Chain in the Cerebellum of Old Mice

**DOI:** 10.3389/fnagi.2018.00032

**Published:** 2018-02-16

**Authors:** Aurel Popa-Wagner, Raluca E. Sandu, Coman Cristin, Adriana Uzoni, Kevin A. Welle, Jennifer R. Hryhorenko, Sina Ghaemmaghami

**Affiliations:** ^1^Department of Neurology, Chair of Vascular Neurology and Dementia, Essen University Hospital, Essen, Germany; ^2^Neurobiology of Aging Group, University of Medicine and Pharmacy Craiova, Craiova, Romania; ^3^School of Medicine, Griffith University, Southport, QLD, Australia; ^4^Institutul Naţional de Cercetare şi Dezvoltare pentru Microbiologie şi Imunologie (Cantacuzino), Bucharest, Romania; ^5^Department of Psychiatry, University of Medicine Rostock, Rostock, Germany; ^6^Department of Biology, University of Rochester, Rochester, NY, United States

**Keywords:** aging, mice, cerebellum, mitochondria, proteins, turnover

## Abstract

Brain structures differ in the magnitude of age-related neuron loss with the cerebellum being more affected. An underlying cause could be an age-related decline in mitochondrial bioenergetics. Successful aging of mitochondria reflects a balanced turnover of proteins involved in mitochondrial biogenesis and mitophagy. Thus, an imbalance in mitochondrial turnover can contribute to the diminution of cellular function seen during aging. Mitochondrial biogenesis and mitophagy are mediated by a set of proteins including MFN1, MFN2, OPA1, DRP1, FIS1 as well as DMN1l and DNM1, all of which are required for mitochondrial fission. Using N15 labeling, we report that the turnover rates for DMN1l and FIS1 go in opposite directions in the cerebellum of 22-month-old C57BL6j mice as compared to 3-month-old mice. Previous studies have reported decreased turnover rates for the mitochondrial respiratory complexes of aged rodents. In contrast, we found increased turnover rates for mitochondrial proteins of the oxidative phosphorylation chain in the aged mice as compared to young mice. Furthermore, the turnover rate of the components that are most affected by aging –complex III components (*ubiquinol cytochrome C oxidoreductase*) and complex IV components (*cytochrome C oxidase*)– was significantly increased in the senescent cerebellum. However, the turnover rates of proteins involved in mitophagy (i.e., the proteasomal and lysosomal degradation of damaged mitochondria) were not significantly altered with age. Overall, our results suggest that an age-related imbalance in the turnover rates of proteins involved in mitochondrial biogenesis and mitophagy (DMN1l, FIS1) in conjunction with an age-related imbalance in the turnover rates of proteins of the complexes III and IV of the electron transfer chain, might impair cerebellar mitochondrial bioenergetics in old mice.

## Introduction

Brain structures differ in the magnitude of age-related neuronal loss. For example, during normal aging in humans and non-human mammals, the number of neurons in the hippocampus remains relatively stable whereas in the cerebellum, a clear age-related neuron loss is observed (Kennard et al., 2013), suggesting that cerebellum-essential tasks show age-related deficits at earlier ages than do hippocampus-essential tasks (Woodruff-Pak and Finkbiner, 1995; Woodruff-Pak et al., 2010).

There is a strong relationship between energy metabolism, aging, and longevity (Lehmann et al., 2008, 2013). For example, calorie restriction correlates with extended life expectancies and fewer age-associated diseases in a wide range of organisms (Heilbronn and Ravussin, 2003). This link is also supported by the free radical hypothesis that links oxidative damage production to energy metabolism and the disposable soma theory which hypothesizes a tradeoff between energy invested in maintenance and reproduction (van Leeuwen et al., 2010). Therefore, mitochondria are important in aging both due to ROS production that contribute to cell damage, and the accumulation of mutations in mitochondrial DNA, which in turn, results in mitochondrial dysfunction (Batlevi and La Spada, 2011; Stefanatos and Sanz, 2017). Thus, comparative studies showed that the stability of mitochondrial DNA, reflected by GC content and mitochondria-associated variables such as resting metabolic rate and body temperature, are determinants (predictors) of mammalian longevity (Lehmann et al., 2008, 2013; Yanai et al., 2016, 2017).

Mitochondria could play a key role in the pathophysiology of aging (Chan, 2006; Navarro et al., 2012; Gonzalez-Freire et al., 2015; Grimm and Eckert, 2017; Herst et al., 2017). In the brain, a recent report showed an age- and brain region-specific differences in mitochondrial bioenergetics in Brown Norway rats (Pandya et al., 2016).

Human cerebral cortex, human cerebellum, and chimpanzee cerebral cortex, each undergo different patterns of age-related gene expression alterations (Fraser et al., 2005). An underlying cause of age-related alterations in gene expression could be an age-related genomic response to altered mitochondrial bioenergetics.

In the rat cerebellum, the number of efficient organelles decreases with advancing age and this may be of critical importance for the proper function of nerve cells when the energy demand is high (Fattoretti et al., 1996). Likewise, mitochondrial metabolic competence, that is, the capacity of mitochondria to provide adequate amounts of energy, declines in the old monkey cerebellum (Bertoni-Freddari et al., 2008).

Recent evidence suggests that complex I activity decreases in the cortex, brainstem and cerebellum of middle aged mice and much more in the cerebellum of the pcd5j mouse, a cerebellar ataxic neurodegeneration model (Pollard et al., 2016). However, the directionality and extent of mitochondrial proteomic alterations underlying the decreased mitochondrial metabolic competence are not clear (Dai et al., 2014; Martin et al., 2014; Stauch et al., 2014; Karunadharma et al., 2015). In the current study, by using ^15^N labeling of proteins in young and old mice, we report highly heterogenous turnover rates for cerebellar mitochondrial proteins.

## Materials and Methods

### Animals and Experimental Design

The subjects of these experiments were young (3-month-old) and aged (18-month-old) male C57BL/6J mice kept under standard laboratory conditions with free access to food and water. For ^15^N-labeling, mice were fed with natural algae for 7 days. After this habituation period, a diet of ^15^N-enriched algae was introduced (*t* = 0) and mice were maintained on this isotopically enriched diet for the remainder of the experiment. At ^15^N-incorporation time points of 0, 1, 2, 4, 8, and 16 days, young (*N* = 2/time point) and aged (*N* = 3/time point) were deeply anesthetized and perfused with buffered saline and the brain tissue was collected. All protocols and procedures were performed in accordance with the guidelines of the Romania Experimental Animals Protection Law and approved by the Animal Experimentation Ethics Board of the University of Medicine and Pharmacy Craiova, Romania.

### Sample Preparation

For protein analysis, dissected cerebellums were homogenized in sample buffer containing 8 M urea, 2 M thiourea, 4% CHAPS, 65 mM DTT, 40 mM Tris and protease inhibitor cocktail (Roth, Karlsruhe, Germany). Total protein was quantified by the Bradford method.

Ten micrograms of each extract was ran on an Invitrogen 4–12% Bis-Tris gel for 5 min at 200V and stained with SimplyBlue SafeStain. Gel bands were diced into small pieces and places in Lowbind tubes. Gel pieces were rinsed with ammonium bicarbonate and acetonitrile and dried in a Speed Vac for 5 min. One hundred microliters of 10 mM DTT in 50 mM ammonium bicarbonate was added to gel pieces and incubated at 55°C for 1 h. Supernatant was removed and 100 μl of 50 mM 2-iodoacetamide in 50 mM ammonium bicarbonate was added to the gel pieces and incubated in the dark for 30 min. After rinsing with ammonium bicarbonate and acetonitrile, the gel pieces were dried in a Speed Vac for 5 min. Trypsin solutions were made by reconstituting Trypsin Gold (100 ug) in 400 ul of 50 mM acetic acid. Trypsin aliquots were diluted to 10 ng/uL with 50 mM ammonium bicarbonate and added to gel pieces. The gels were incubated at room temperature for 1 h for rehydration and then placed at 37°C overnight. Solutions were transferred into a clean 0.5 ml Lobind Tube and 50% ACN/0.1% TFA was added to cover gel pieces and shaken for 25 min. Extracted supernatants were pooled and freeze dried. The samples were resuspended in 50 μL of 0.1% TFA, and proceeded to mass spectrometry analysis.

### LC-MS/MS Analysis

Six microliters of each sample was analyzed on a Q Exactive Plus mass spectrometer (Thermo Scientific) coupled with an Easy nLC-1000 pump (Thermo Scientific). Columns were hand-pulled using 100 um fused silica, which was packed with 30 cm of 1.8 um, 120 Angstrom C18 beads (Sepax). Mobile phase A was 0.1% formic acid in water, and mobile phase B was 0.1% formic acid in acetonitrile. Peptides were separated using a gradient of 8–30% B over 145 min, 30–50% B over 10 min, and 50–70% B over 4 min, holding at 70% B for 5 min. The gradient returned to 0% B in 4 min, and held there for 10 min to prepare for the next injection. The flow rate throughout the run was held at 300 uL/min. A data-dependent top 10 MS2 method was used for peptide detection and fragmentation. For each cycle, one full scan from m/z 400–1700 was acquired in the Orbitrap at a resolution of 70,000 at m/z = 200, with an AGC target of 3e6, and a maximum injection time of 50 ms. After each full scan, the top 10 most intense ions were selected for fragmentation in the HCD cell, followed by detection in the Orbitrap at a resolution of 35,000 at m/z = 200. The AGC target was set to 1e5, with a maximum injection time of 150 ms, an isolation width of 1.5 Da, and a normalized collision energy of 27. A dynamic exclusion window of 25 s was used, while excluding unassigned, +1, and greater than +5 charge states. The polysiloxane 445.12003 m/z was used for lockmass. For Stable Isotope Labeling in Cell Culture (SILAC) LC-MS/MS analysis on the TMT-labeled, un-mixed samples, all settings remained the same except for the isolation width, which was adjusted up to 1.0 Da, with an offset of 0.3.

### Data Analysis

MS/MS peaklists were extracted using the program PAVA from the LC-MS/MS raw data files corresponding to the unlabeled tissues. Peptide identification was conducted by Protein Prospector against the Mus Musculus Uniprot database (downloaded June 27, 2016). To this database, a randomized version was concatenated to allow determination of false discovery rates. The search parameters were the following: species = Mus musculus; enzyme specificity = trypsin; allowed missed cleavages = 1; fixed modification = carbamidomethylation; variable modifications = acetylation on protein N terminus, glutamine on peptide N-terminal to glutamic acid, methionine loss from protein N terminus, methionine loss from protein N terminus and acetylation, methionine oxidation; maximal number of variable modifications = 2; parent mass tolerance = 50 ppm; fragment mass tolerance = 0.6 Da; peptide expectation cutoff = 0.05. The match of sequences from the decoy database (normal + random) indicated that the false discovery rates for this expectation cutoff value were less than 0.2% for all analyzed datasets. A complete list of protein identifications and supporting statistics are provided in **Supplementary Table S1**.

Using the Protein Prospector database search results, the following information was gathered for each peptide ion with an expectation value less than 0.05: monoisotopic mass to charge ratio (m/z), charge (z), retention time (RT), assigned protein and sequence. The data were tabulated as a text file. Using the program PAVA(1), MS1 spectra were extracted from all raw data files obtained from the labeling time-course. The MS1 spectra were centroided and the resulting peaklists were used for further analysis. Using a script written in Java (*mssplice*) (Guan et al., 2011), the following set of analyses were sequentially conducted for each tabulated peptide obtained from the database search:

(1) Numbers of nitrogen atoms were determined for each peptide sequence.
(2) The possible set of isotopic m/z values for each peptide was determined, assuming a range of 0.0–1.0 ^15^N incorporation ratios.
(3) Using a series of overlapping 30-s RT windows, ranging from 2 min prior to 2 min passed the experimentally determined RT, MS1 spectra ranging from the monoisotopic m/z to the maximum possible isotopic m/z (assuming 100% ^15^N incorporation) were collected and averaged. The total intensity of all possible peptide isotopic m/z values were summed and plotted as a function of RT to generate a chromatogram for each peptide. The chromatogram was fitted with a Gaussian function to determine the peak width. MS1 spectra within the determined RT peak width and the m/z window were summed and the aggregated spectra were used for further analysis.
(4) The extracted MS1 peptide spectra were fitted with a probabilistic combinatorial isotopic distribution model that considers the spectra as the sum of two isotopic populations (labeled and unlabeled). The ^15^N enrichment of the unlabeled population was set as a constant to its natural abundance. The ^15^N enrichment of the labeled population and the ratio of labeled and unlabeled populations were considered as the two fitted variables. Least-square fitting to the model was used to obtain the following information: the fraction ^15^N incorporation of the labeled population, the ratio of labeled and unlabeled populations, goodness of fits (coefficient of determination – *R*^2^) to predicted isotopomer distributions for labeled and unlabeled populations. Only spectra that had *R*^2^ values of greater than 0.8 for both populations were used for further analysis.
(5) For each peptide ion where more than four unique time-points passed the above criteria, the fraction labeled values were fitted to a single exponential equation to determine the clearance rate.
(6) Peptide level labeled population measurements were aggregated for each homologous group of proteins. Peptides with sequences that were shared among multiple homologous groups were not considered.
(7) The aggregated data were fitted with a single exponential equation to determine protein clearance rates. A complete list of measured turnover rates are provided in the **Supplementary Table S1**.

## Results

Proteome-wide analyses of turnover by mass spectrometry have been greatly propelled by the development of SILAC – a set of standardized protocols, reagents and analysis tools aimed at quantifying the incorporation of ^15^N/^13^C labeled amino acids into proteins (Ong and Mann, 2007). Conducting dynamic (i.e., time-resolved) SILAC experiments are now fairly routine and have enabled global analyses of protein turnover in a number of cellular systems (Cambridge et al., 2011; Schwanhausser et al., 2011). In a recent study (Price et al., 2010), we modified a ^15^N labeling strategy developed by Wu et al. (2004) to measure *in vivo* turnover rates in various mouse tissues. The procedure involves the complete isotopic labeling of blue green algae (*Spirulina platensis*) with ^15^N labeled salts and utilizing it as a dietary source of nitrogen for mice. By quantifying mass shifts and isotopomer distributions, we are able to obtain turnover rates for thousands of proteins across multiple tissues. In this study, we measured rate constants for fractional isotopic labeling to analyze age-induced changes in global rates of degradation in mice. Thus, the measured rate constants correspond to the first order rate constant for degradation. Differences in synthesis rates do not influence this rate constant (Claydon and Beynon, 2012).

The assembly and maintenance of respiratory complexes is central to both successful aging and protection from age-related diseases. Thus, an imbalance in mitochondrial turnover can contribute to a loss of function seen during aging.

**FIGURE 1:**
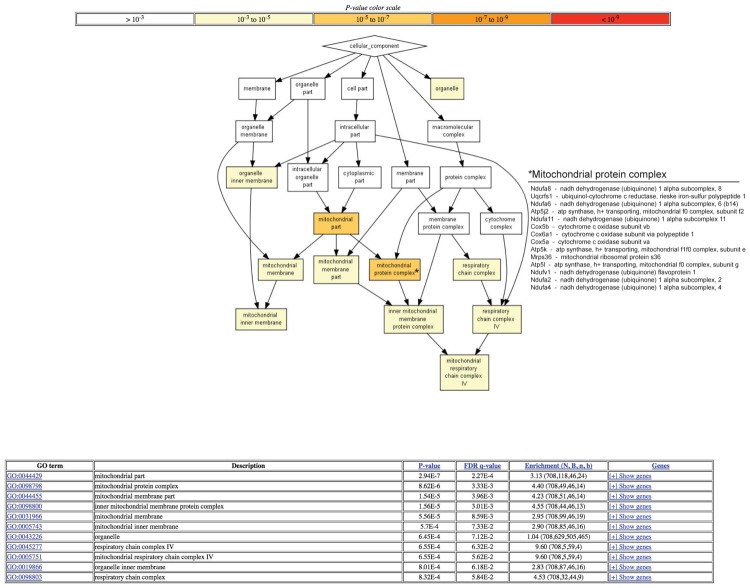
Gene ontology (GO) analysis of proteins with enhanced degradation in old mice. Log2 ratios of degradation rates between old and young mice cerebellum samples were measured (see Materials and Methods). A hierarchical GO enrichment analysis was conducted for proteins with enhanced degradation in the old mice. The statistical significance (*P*-value) of gene enrichment of differentially expressed genes within each GO accession is indicated by the color shading. The hierarchical relationship between accessions is indicated by arrows. The analysis was conducted using the algorithm GOrilla (2). The table provides additional information about each accession, including the false discovery rate and enrichment level. This figure is limited to the cellular component GO category. The complete results of the GO analysis are tabulated in **Supplementary Table S2**. A list of proteins belonging to the GO term “mitochondrial protein complex” that are enriched among the subset of proteins with enhanced degradation rates in old mice is provided.

A hierarchical gene ontology (GO) enrichment analysis of proteins with enhanced degradation using the algorithm GOrilla (Eden et al., 2009) in old mice revealed most significant changes in the mitochondrial part and in particular in the mitochondrial protein complex (**Figure 1**). The complete results of the GO analysis are tabulated in **Supplementary Table S2**.

**FIGURE 2:**
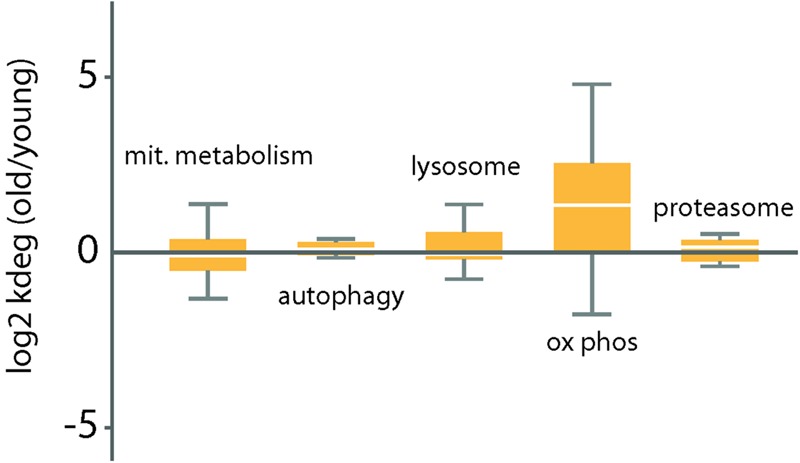
Distribution of log2 ratios of degradation rates between old and young mice for a select group of functional categories related to protein homeostasis. Log2 ratios of degradation rates between old and young mice cerebellum samples were measured (see Materials and Methods). Box plots indicate the distribution of log_2_ ratios degradation rates for each category. The box indicates the interquartile range (IQR) and the line indicates the median. Far outliers (>1.5^∗^IQR) were excluded.

Mitochondrial mass is modulated during the lifespan of the cell, in accordance with the physiological state. This modulation involves mitochondrial biogenesis including metabolites supply, fusion and fission but also and mitochondrial degradation, including autophagy, lysosome and proteasome processes. However, of these, only the turnover rates of proteins of the oxidative phosphorylation complexes were significantly changed in the cerebellum of the old mice (**Figure 2**). In particular, we noted significantly increased degradation rates in the components of the complex I (NADH dehydrogenase), complex III (ubiquinol cytochrome C oxidoreductase), complex IV (cytochrome C oxidase), and complex V (ATP synthase) in the cerebellum of old mice. For complex 2, a single subunit was quantifiable.

In the following, we give an account of changes in the degradation rates of specific proteins of the oxidative phosphorylation complexes (**Table 1**). The dynamic balance of fusion and fission of mitochondria determines their morphology and allows their immediate adaptation to energetic needs. It also preserves mitochondria functionality and morphology by restoring or removing damaged organelles or precipitates cells in apoptosis in cases of severe defects. Thus, we noted that the turnover rate of dynamin-1-like protein (Dmn1l), a protein that mediates mitochondria fission, was three-fold increased in the cerebellum of old mice. However, the turnover rate of dynamin 1,0 (Dnm1), a protein also involved in mitochondrial fission, did not change significantly with increasing age. Finally, the turnover rate of mitochondrial fission 1 protein (Fis1), also involved in mitochondrial fission, was decreased two-fold in the cerebellum of the old mice.

**Table 1:** Changes in the degradation rates of specific proteins of the oxidative phosphorylation complexes.

Uniprot	Name	Young kdeg	Old kdeg	Log_2_o/y ratio
**Complex 1**				
Q9CQZ6	NADH DEHYDROGENASE (UBIQUINONE) 1 BETA SUBCOMPLEX 3	0.038	0.692	4.153
Q91YT0	NADH DEHYDROGENASE (UBIQUINONE) FLAVOPROTEIN 1	0.025	0.294	3.534
Q9CQ54	NADH DEHYDROGENASE (UBIQUINONE) 1, SUBCOMPLEX UNKNOWN, 2	0.016	0.150	3.167
Q9CQZ5	NADH DEHYDROGENASE (UBIQUINONE) 1 ALPHA SUBCOMPLEX, 6 (B14)	0.023	0.171	2.867
Q99LY9	NADH dehydrogenase (ubiquinone) iron-sulfur protein 5	0.017	0.127	2.856
Q9D8B4	NADH DEHYDROGENASE (UBIQUINONE) 1 ALPHA SUBCOMPLEX 11	0.024	0.136	2.500
Q9CQ75	NADH DEHYDROGENASE (UBIQUINONE) 1 ALPHA SUBCOMPLEX, 2	0.022	0.088	1.957
Q9CQJ8	NADH DEHYDROGENASE (UBIQUINONE) 1 BETA SUBCOMPLEX, 9	0.010	0.039	1.940
Q62425	NADH DEHYDROGENASE (UBIQUINONE) 1 ALPHA SUBCOMPLEX, 4	0.033	0.126	1.922
Q9DQ5	NADH DEHYDROGENASE (UBIQUINONE) 1 ALPHA SUBCOMPLEX, 8	0.014	0.051	1.861
Q9Z1P6	NADH DEHYDROGENASE (UBIQUINONE) 1 ALPHA SUBCOMPLEX, 7 (B14.5A)	0.047	0.112	1.248
Q9CPP6	NADH DEHYDROGENASE (UBIQUINONE) 1 ALPHA SUBCOMPLEX, 5	0.053	0.104	0.958
Q9CR61	NADH DEHYDROGENASE (UBIQUINONE) 1 BETA SUBCOMPLEX, 7	0.012	0.022	0.906
P52503	NADH DEHYDROGENASE (UBIQUINONE) FE-S PROTEIN 6	0.034	0.058	0.761
Q9ERS2	NADH DEHYDROGENASE (UBIQUINONE) 1 ALPHA SUBCOMPLEX, 13	0.018	0.027	0.522
Q9DCS9	NADH DEHYDROGENASE (UBIQUINONE) 1 BETA SUBCOMPLEX, 10	0.016	0.022	0.402
Q9D6J5	NADH DEHYDROGENASE (UBIQUINONE) 1 BETA SUBCOMPLEX 8	0.021	0.022	0.067
Q9D6J6	NADH DEHYDROGENASE (UBIQUINONE) FLAVOPROTEIN 2	0.033	0.033	0.026
Q9DCT2	NADH DEHYDROGENASE (UBIQUINONE) FE-S PROTEIN 3	0.022	0.014	-0.657
Q8K3J1	NADH DEHYDROGENASE (UBIQUINONE) FE-S PROTEIN 8	0.044	0.023	-0.943
Q9DC69	NADH DEHYDROGENASE (UBIQUINONE) 1 ALPHA SUBCOMPLEX, 9	0.040	0.021	-0.932
Q99LC3	NADH DEHYDROGENASE (UBIQUINONE) 1 ALPHA SUBCOMPLEX 10	0.102	0.022	-2.184
Q7TMF3	NADH DEHYDROGENASE (UBIQUINONE) 1 ALPHA SUBCOMPLEX, 12	0.207	0.037	-2.470
Q9DC70	NADH DEHYDROGENASE (UBIQUINONE) FE-S PROTEIN 7	0.240	0.028	-3.079
**Complex II**				
Q9CZB0	SUCCINATE DEHYDROGENASE COMPLEX, SUBUNIT C, INTEGRAL MEMBRANE PROTEIN	0.062	0.248	1.977
**Complex III**				
Q9CQ69	UBIQUINOL-CYTOCHROME C REDUCTASE, COMPLEX III SUBUNIT VII	0.003	5.456	10.608
P99028	UBIQUINOL-CYTOCHROME C REDUCTASE HINGE PROTEIN	0.029	0.166	2.511
Q9CR68	UBIQUINOL-CYTOCHROME C REDUCTASE, RIESKE IRON-SULFUR POLYPEPTIDE 1	0.018	0.082	2.124
Q921G7	ELECTRON TRANSFERRING FLAVOPROTEIN, DEHYDROGENASE	0.037	0.080	1.085
Q9D855	UBIQUINOL-CYTOCHROME C REDUCTASE BINDING PROTEIN	0.187	0.060	-1.632
Q9DB77	UBIQUINOL CYTOCHROME C REDUCTASE CORE PROTEIN 2	0.023	0.012	-0.860
Q9CZ13	UBIQUINOL-CYTOCHROME C REDUCTASE CORE PROTEIN 1	0.012	0.011	-0.050
**Complex IV**				
P48771	CYTOCHROME C OXIDASE, SUBUNIT VIIA 2	0.030	0.601	4.304
P62897	CYTOCHROME C, SOMATIC	0.009	0.144	3.895
Q9CPQ1	CYTOCHROME C OXIDASE, SUBUNIT VIC	0.017	0.119	2.795
P12787	CYTOCHROME C OXIDASE, SUBUNIT VA	0.017	0.123	2.790
P19536	CYTOCHROME C OXIDASE, SUBUNIT VB	0.020	0.078	1.962
P43024	CYTOCHROME C OXIDASE, SUBUNIT VI A, POLYPEPTIDE 1	0.047	0.129	1.448
Q9D0M3	CYTOCHROME C-l	0.085	0.057	0.569
P19783	CYTOCHROME C OXIDASE SUBUNIT IV ISOFORM 1	0.035	0.018	-0.970
**Complex V**				
P56382	ATP SYNTHASE, H+TRANSPORTING, MITOCHONDRIAL Fl COMPLEX, EPSILON SUBUNIT	0.008	0.745	6.411
P03930	ATPASE SUBUNIT 8, FO COMPLEX	0.221	6.149	4.798
Q06185	ATP SYNTHASE, H+TRANSPORTING, MITOCHONDRIAL FIFO COMPLEX, SUBUNIT E	0.008	0.075	3.099
P97450	ATP SYNTHASE, H+ TRANSPORTING, MITOCHONDRIAL FO COMPLEX, SUBUNIT F	0.043	0.257	2.557
P56135	ATP SYNTHASE, H+TRANSPORTING, MITOCHONDRIAL FO COMPLEX, SUBUNIT F, ISOFORM 2	0.022	0.107	2.229
Q9CPQ8	ATP SYNTHASE, H+TRANSPORTING,	0.022	0.064	1.519
	MITOCHONDRIAL FO COMPLEX, SUBUNIT G			
Q03265	ATP SYNTHASE, H+TRANSPORTING, MITOCHONDRIAL Fl COMPLEX, ALPHA SUBUNIT, ISOFORM 1	0.014	0.022	0.592
P56480	ATP SYNTHASE, H+TRANSPORTING MITOCHONDRIAL Fl COMPLEX, BETA SUBUNIT	0.016	0.019	0.246
Q9CQQ7	ATP SYNTHASE, H+TRANSPORTING, MITOCHONDRIAL FO COMPLEX, SUBUNIT B, ISOFORM 1	0.013	0.014	0.119
Q9D3D9	ATP SYNTHASE, H+ TRANSPORTING, MITOCHONDRIAL Fl COMPLEX, DELTA SUBUNIT	0.008	0.009	0.186
Q9DCX2	ATP SYNTHASE, H+TRANSPORTING, MITOCHONDRIAL FO COMPLEX, SUBUNIT D	0.012	0.011	-0.180
Q9DB20	ATP SYNTHASE, H+TRANSPORTING, MITOCHONDRIAL Fl COMPLEX, 0 SUBUNIT	0.012	0.010	-0.261
Q91VR2	ATP SYNTHASE, H+TRANSPORTING, MITOCHONDRIAL Fl COMPLEX, GAMMA POLYPEPTIDE 1	0.018	0.014	-0.410

Aging of the mammalian brain is associated with decreased electron transfer in mitochondria isolated from old animals and a continuous decrease of the capacity to produce ATP by oxidative phosphorylation (Boveris and Navarro, 2008). Decreased ATP production could be due to either a failure of the biochemical pathway to supply mitochondria with pyruvate and reducing power or a decreased efficiency of electron transfer in mitochondria due to age-related changes in the components of the oxidative chain. We found that turnover rates in the enzymes and proteins required for mitochondria supply with oxidative phosphorylation substrates did not change significantly with age (**Table 1**).

**FIGURE 3:**
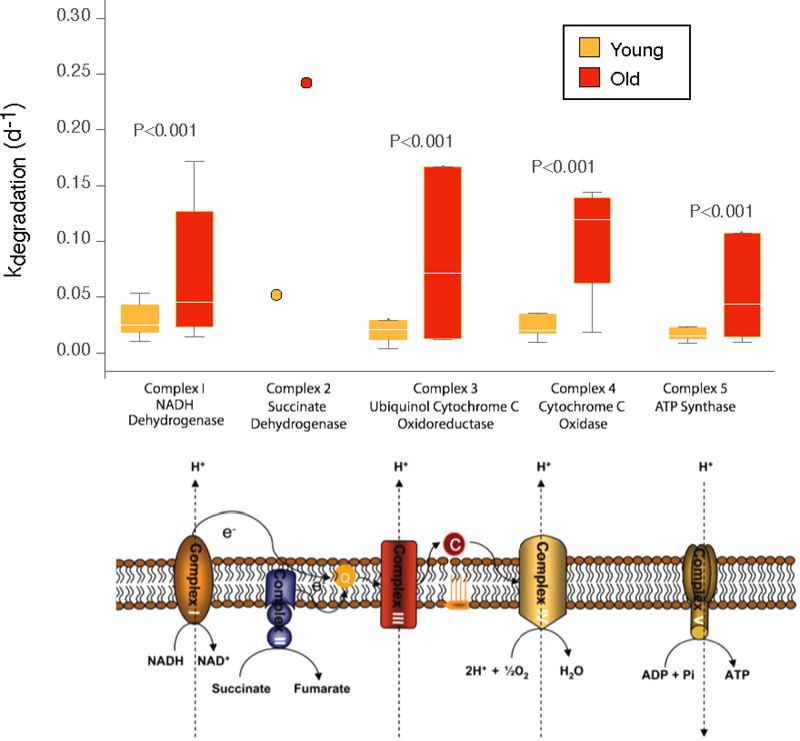
Distribution of degradation rates for old and young mice for components of the mitochondrial transport chain. Box plots indicate the distribution of degradation rates for all subunits quantified for each complex. The box indicates the interquartile range (IQR) and the line indicates the median. Far outliers (>1.5^∗^IQR) were excluded. *P*-values for comparisons between old and young mice were conducted by two-sided Mann–Whitney *U* test, considering values from different subunits as independent measurements of k_degradation_ (d^-1^) for a given complex. For complex 2, a single subunit was quantifiable, and the data is shown as that single measurement rather than a distribution.

Previous studies have shown that mitochondria isolated from brain, liver, heart, and kidney of old mice showed decreased electron transfer activity in complexes I and IV, whereas complexes II and III were largely unaffected (Navarro and Boveris, 2004, 2005, 2007). In terms of turnover rates, we found a significant increase in the turnover rates of the complex IV components (cytochrome C oxidase) and to a lesser extent in the complex III (ubiquinol cytochrome C oxidoreductase) whereas turnover rates in the complexes I, II, and V were largely unaffected by increasing age (**Figure 3**). Within complex IV, increases in the degradation rate were found for cytochrome oxidase subunit VIIA (4.3-fold) of the old mice cerebellum, followed by cytochrome C, an electron carrier protein that transfers electrons to the cytochrome oxidase complex and cytochrome oxidase subunit VIC and cytochrome oxidase subunit VA (both, 2.7-fold) and to a lesser extent cytochrome oxidase subunit VB (1.9-fold). One protein showed, nevertheless, decreased turnover rates in the mitochondria from old cerebellum, i.e., cytochrome oxidase subunit IV (-0.97).

The second largest changes in turnover rates of proteins of the mitochondrial respiratory chain were noted for complex III, and especially for ubiquinol cytochrome C reductase subunit VII (10.6-fold), ubiquinol cytochrome C reductase hinge protein (2.5-fold) and ubiquinol cytochrome C reductase Rieske iron-sulfur polypeptide 1 (2.12-fold). Negative turnover rates in the old cerebellum were found for ubiquinol cytochrome C reductase binding protein (-1.63-fold) and ubiquinol cytochrome C reductase core protein (-0.86-fold).

## Discussion

Age-related changes in cerebellar mitochondria are reflected in alterations of their morphology, function, and biochemical properties. Further, an imbalance in mitochondrial turnover can contribute to a loss of function seen during aging. In this work, using a different labeling strategy of mitochondrial proteins, we report increased turnover rates for mitochondrial proteins of the complexes III and IV of the electron transport chain in the cerebellum of old mice. Nevertheless, we noted that complexes III and IV were more dramatically affected in comparison with complexes I, II, and V.

Results on the mitochondrial proteins turnover with increasing age have yielded conflicting results (Ingram and Chakrabarti, 2016). A slower rate of mitochondrial turnover with increasing age caused by reduced mitochondrial biogenesis and/or inefficient mitochondrial degradation has been documented (Terman et al., 2010; Martin et al., 2014). Further, by quantitative proteomics there was an age-dependent decrease in the levels of proteins involved in mitochondrial function, electron transport chain, citric acid cycle, and fatty acid metabolism as well as an increased abundance of proteins involved in glycolysis and oxidative stress response (Dai et al., 2014).

A recent study addressed directly the turnover rates of mitochondrial proteins across various tissues in young and old mice. Thus, using metabolic [^2^H_3_]-leucine heavy isotope labeling, it was found that the turnover rates were highly heterogeneous between subunits of the respiratory complexes (Karunadharma et al., 2015).

As it has been shown for muscle, the changes in protein homeostasis in the aged mitochondria suggest a breakdown of coordination of biosynthesis and protein recycling which might result in increased degradation rates for some components of the electron transfer chain. Thus, the loss of stoichiometry and lower mitochondrial efficiency due to the elevation of mitochondrial protein expression, particularly in complex I, is indicative of lack of a coordinated response of the mitochondrial proteome to age-induced stress in susceptible tissue (Kruse et al., 2016).

Functional mitochondria is maintained by timely replacement of its components, in particular the proteins of the electron transfer chain, that are exposed to aggressive ROS generated during oxidative phosphorylation (Wang et al., 2013). Therefore, an increased turnover rate for components of the respiratory chain in the old cerebellum might also reflect an increased need to replace worn out components of the electron transfer chain which accumulate with increasing age in long-lived cells like neurons (Wang et al., 2013).

Mitochondria are extremely dynamic organelles engaged in a constant process of fission and fusion that constantly rejuvenate mitochondria (Detmer and Chan, 2007). Ideally, successful aging of mitochondria shall reflect a balance between mitochondria biogenesis and mitophagy. This process is mediated by a set of proteins including (i) mitofusins 1 and 2 (Mfn1, Mfn2) which mediate the outer membrane fusion, (ii) optic atrophy 1 (OPA1) which mediates inner membrane fusion, (iii) dynamin-related protein 1 (Drp1) and Fis1 as well as (iv) dynamin-1-like protein (Dmn1l) and dynamin 1,0 (Dnm1), all being required for mitochondria fission (Detmer and Chan, 2007; Batlevi and La Spada, 2011).

Proteins that control mitochondrial fusion/fission dynamics are ubiquitous. Therefore, their disturbances are associated with several diseases. This is particularly relevant for the brain because of the constant need for high levels of energy in neuronal cells. Age-related degeneration of cerebellar Purkinje cells has been linked to various age-related disabilities including dysfunction of postural control, cerebellar ataxia, essential tremor, Alzheimer’s disease and Lewy Body disease (Di Bella et al., 2010; Zhang et al., 2010). Clinically, mitochondrial dysfunction has emerged as a potential mechanism underlying Parkinson’s disease (PD). More specifically, complexes I and II deficiency has been reported in homogenates of postmortem *substantia nigra* samples of PD patients (Schapira et al., 1990; Grünewald et al., 2016). Further, familial PD cases were found to harbor mutations in proteins involved in the removal of damaged mitochondria (Kumar et al., 2011).

Current evidence suggests that unbalanced mitochondrial dynamics may contribute to Huntington disease (HD), too (Oliveira and Lightowlers, 2010; Costa and Scorrano, 2012). Thus, HD patients exhibit elevated mRNA and protein levels of the fission-associated genes, DRP1 and FIS1, together with decreased levels of mitofusins in striatal and cortical regions (Kim et al., 2010; Shirendeb et al., 2012). It should be noted that *Drp1* deletion in the cerebellum results in (i) mitochondrial swelling, increased oxidative damage, (ii) ubiquitination of mitochondrial proteins, (iii) accumulation of autophagy markers and, (iv) impaired activity of the complex IV activity, ultimately leading to neurodegeneration (Kageyama et al., 2012).

Similarly, increased mitochondrial fragmentation and decreased levels for Mfn1 and Mfn2 in cultured cortical neurons and cortical neurons derived from a newly developed HD mouse model (BACHD) have been reported. Furthermore, increased *Drp1* and *Fis1* mRNA levels were found in HD mice (Shirendeb et al., 2012). Of note, germline mutations in *Mfn2* cause Charcot–Marie–Tooth hereditary-like neuropathy type 2A (CMT2A), while germline mutations in OPA1 causes the autosomal dominant optic atrophy (ADOA) which is the most common genetic cause of optic atrophy in humans (Knott and Bossy-Wetzel, 2008).

Mitochondrial fission is also important for neuronal function, as dominant-negative *Drp1* mutation can cause a lethal infantile neurodegenerative phenotype (Waterham et al., 2007). Thus, *Drp1* knock-out mice display embryonic lethality, increased mitophagy and dilated cardiomyopathy, associated with increased levels of necrotic cell death, as well as abnormal brain development and failure to develop synapses (Ishihara et al., 2009; Wakabayashi et al., 2009; Song et al., 2015). Mitochondrial fission has also been linked to apoptotic cell death of neurons (Knott and Bossy-Wetzel, 2008). On the other hand, inhibition of mitochondrial fission with dominant-negative *Drp1* or by *Fis1* RNA knock-down, prevents mitophagy and results in accumulation of damaged mitochondria (Twig et al., 2008).

Mitophagy has been linked to aging, as impaired macroautophagy over time promotes mitochondrial dysfunction associated with the aging process. Further, an impaired macroautophagy over time promotes mitochondrial dysfunction associated with the aging process (Batlevi and La Spada, 2011).

It has been reported that aging is associated with an increased level of oxidized proteins and an imbalance in proteostasis due to impairment of the ubiquitin/proteasome system (Maharjan et al., 2014; Gonzalez-Freire et al., 2015). However, our results indicate that the turnover rates of proteins involved in mitophagy, proteasome and lysosomal degradation of damaged mitochondria are not significantly changed in the cerebellum of old mice.

### Study Limitations

In this study, we report age-related changes in the degradation rate of proteins of the complexes III and IV of the electron transport chain in the old cerebellum. However, since the degradation rates of proteins are specific for different brain regions, the full significance of increased degradation rates for cerebellar mitochondrial proteins shall be clarified by additional studies on the other brain regions.

## Conclusion

Previous studies have reported decreased turnover rates for mitochondrial proteins with increasing age in rodents. In the current study, by using N15 labeling, we found highly heterogeneous turnover rates for mitochondrial proteins, but contrary to prior findings, our results do not indicate decreased turnover rates for mitochondrial proteins in the aged mouse cerebellum. On the contrary, we found significantly increased turnover rates for several proteins of the electron transport chain, especially for components of the complex III (ubiquinol cytochrome C oxidoreductase) and complex IV (cytochrome C oxidase). Our results also indicate that the turnover rates of proteins involved in mitophagy are not significantly altered in the cerebellum of old mice.

## Author Contributions

All authors listed have made a substantial, direct and intellectual contribution to the work, and approved it for publication.

## Conflict of Interest Statement

The authors declare that the research was conducted in the absence of any commercial or financial relationships that could be construed as a potential conflict of interest.

## Supplementary Material

TABLE S1 A complete list of protein identifications and measured turnover rates for the young and aged mouse cerebellum.

TABLE S2 The complete results of the GO analysis for the young and aged mouse cerebellum.
